# Relapsing bronchopneumonia due to community-associated methicillin-resistant *Staphylococcus aureus*: a case report

**DOI:** 10.1186/s12879-024-09268-2

**Published:** 2024-04-04

**Authors:** Sho Shimada, Tetsuo Yamaguchi, Satsuki Mikoshiba, Kazuaki Sato, Takahiro Mitsumura, Kohji Komori, Takashi Yamana, Yuki Iijima, Rie Sakakibara, Sho Shibata, Takayuki Honda, Tsuyoshi Shirai, Tsukasa Okamoto, Haruhiko Furusawa, Tomoya Tateishi, Yasunari Miyazaki

**Affiliations:** 1 Department of Respiratory Medicine, Tokyo Medical and Dental University (TMDU), 1-5-45 Yushima, Bunkyo-Ku, Tokyo, 113-8519 Japan; 2 Department of Microbiology and Infectious Diseases, Toho University School of Medicine, 5-21-16 Omorinishi, Ota-Ku, Tokyo, 143-8540 Japan; 3 Department of Respiratory Medicine, National Hospital Organization Disaster Medical Center, 3256 Midoricho, Tachikawa, Tokyo, 190-0014 Japan; 4 Department of Respiratory Medicine, Soka Municipal Hospital, 2-21-1 Soka, Soka-Shi, Saitama, 340-8560 Japan; 5 Department of Respiratory Medicine, Respiratory Center, Toranomon Hospital, 2-2-2 Toranomon, Minato-Ku, Tokyo, 105-8470 Japan

**Keywords:** Community-associated methicillin-resistant *Staphylococcus aureus*, Sequence type 1, Relapsing bronchopneumonia, Trimethoprim/sulfamethoxazole

## Abstract

**Background:**

The emergence of community-associated methicillin-resistant *Staphylococcus aureus* (CA-MRSA) has increased the incidence of community-onset MRSA infection. Respiratory tract infections caused by MRSA has been noted for their severity; however, repeated relapses that require extended antibiotic therapy are rare.

**Case presentation:**

We report a case of relapsing bronchopneumonia caused by CA-MRSA in a 56-year-old man. The patient responded to antibiotics, but repeatedly relapsed after stopping treatment. MRSA was consistently isolated from airway specimens during each relapse. Extended oral antibiotic treatment with trimethoprim/sulfamethoxazole (TMP/SMX) for 6 months achieved infection control. Whole-genome sequencing of the isolated strain revealed that the causative agent was sequence type (ST)1/staphylococcal cassette chromosome mec (SCC*mec*) type IVa, a clone that is rapidly increasing in Japan.

**Discussion and conclusions:**

This patient had an unusual course of MRSA bronchopneumonia with repeated relapses. Although the choice of antibiotics for long-term use in MRSA respiratory tract infections has not been well established, TMP/SMX was effective and well tolerated for long-term therapy in this case. The clinical course of infections related to the rapid emerging clone, ST1/SCC*mec* type IVa warrants further attention.

## Background

The emergence of community-associated methicillin resistant *Staphylococcus aureus* (CA-MRSA) worldwide in the past few decades has led to an increase in the incidence of the community-onset infections. Although the spread of CA-MRSA infection may lead to an increase in the number of refractory community-onset infections, the pathogenic traits of newly emerging CA-MRSA clones in Japan are not well characterized.

MRSA respiratory tract infections are noted for their severity; however, cases requiring long-term antimicrobial therapy are rare. We describe a case of relapsing bronchopneumonia caused by CA-MRSA that required long-term antibiotic treatment. Whole-genome sequencing revealed that the causative strain was an emerging CA-MRSA clone that has recently spread in Japan [1].

## Case presentation

**Fig. 1:**
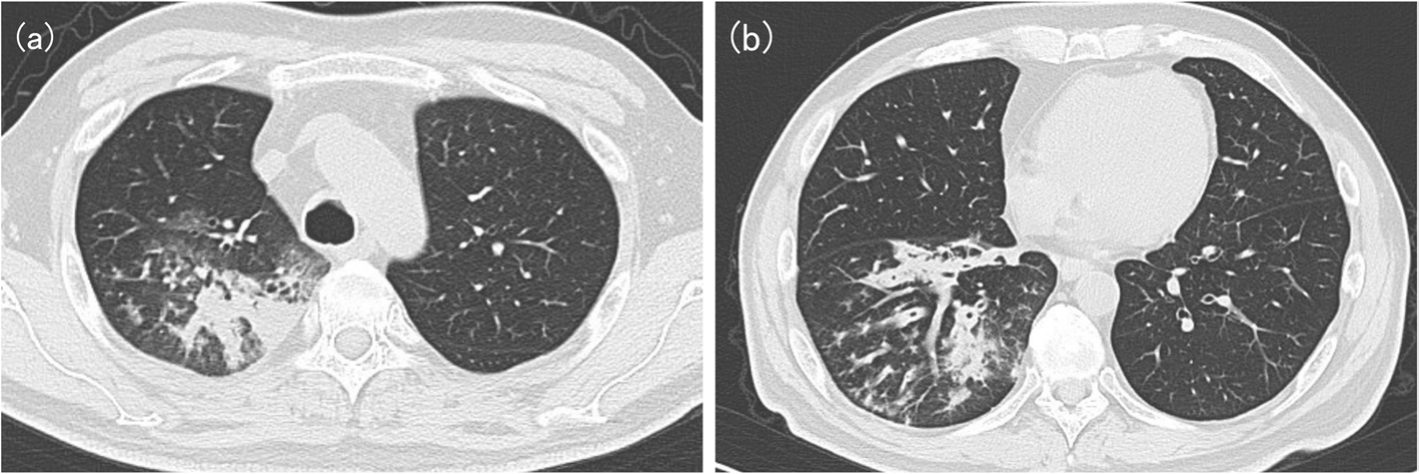
CT showing bronchial wall thickening and focal consolidation at right **a** upper and **b** lower lobes

A 56-year-old man with hepatitis C, diabetes, and mild chronic obstructive pulmonary disease (COPD) due to active smoking (Brinkman index: 200) was referred to our hospital with a one-month history of occasional bloody sputum. He had been treated for right lower lobe pneumonia in the outpatient clinic 2 years previously. Initial blood tests showed a white blood cell (WBC) count of 12,300/μL and a C-reactive protein (CRP) level of 3.5 mg/L. Chest computed tomography (CT) revealed granular opacities, patchy consolidation along the airways, and bronchial wall thickening in the right upper and lower lobes. No signs of emphysema, bronchiectasis, or lung abscess was observed (Fig. 1). Sputum culture confirmed MRSA and mycobacterial culture was negative. At a one-week follow-up, the patient returned with worsening symptoms. His CRP level had increased to 47.5 mg/L. Based on the drug susceptibility test results (Table 1), oral trimethoprim/sulfamethoxazole (TMP/SMX) 160/800 mg twice daily was initiated and continued for 2 weeks, leading to rapid improvement in his symptoms and radiological findings. However, symptoms of productive cough relapsed with worsening radiological findings 2 weeks after TMP/SMX discontinuation.

**Table 1:** Genome characteristics and drug susceptibility of TUM20898

Isolates	Genotype			Virulence genes	Resistance genes	Antimicrobial Susceptibility (μg/mL)								
	SCC*mec*	MLST	spa			EM	CLDM	LVFX	MINO	TMP/SMX	ABK	VCM	LZD	TEIC
TUM20898	IVa	ST1	t1784	*aur, splA, splB, splE, hlgA, hlgB, hlgC, lukD, lukE, seh sak, scn*	*aac*(6')*-aph*(2'')*, erm*(A)	> 4	≤ 0.25	> 4	≤ 2	≤ 1/19	≤ 1	1	2	≤ 1

*Abbreviations*: *ABK* arbekacin, *EM* erythromycin, *CLDM* clindamycin, *LVFX* levofloxacin, *LZD* linezolid, *MINO* minocycline, *MLST* multilocus sequence typing, *SCCmec* staphylococcal cassette chromosome mec, *TMP/SMX* trimethoprim/sulfamethoxazole, *TEIC* teicoplanin, *VCM* vancomycin

**Fig. 2:**
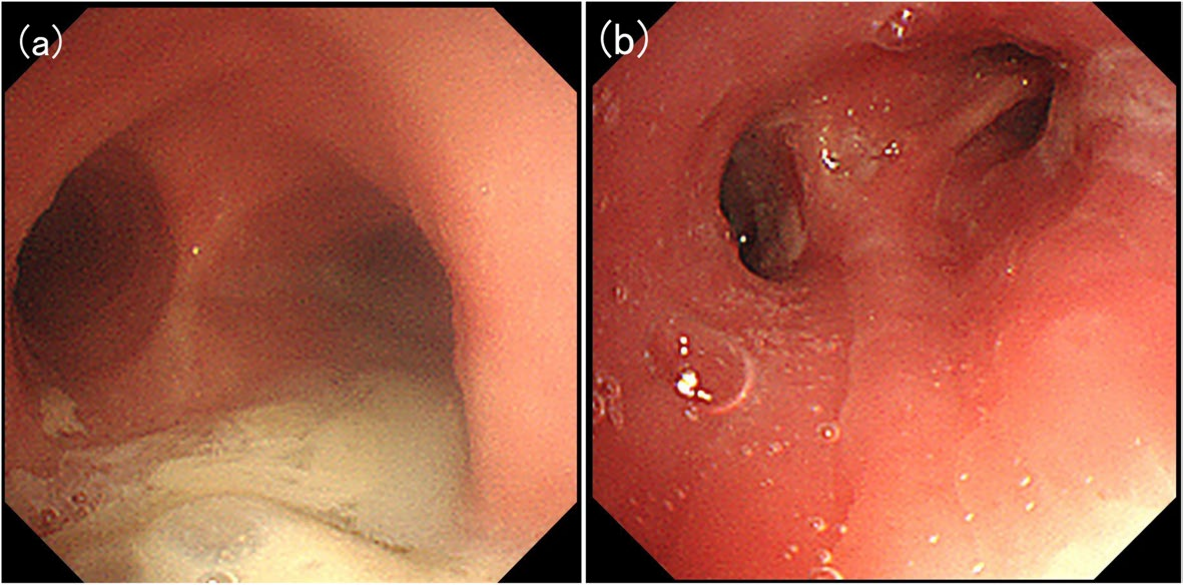
Bronchoscopic image showing sputum retention and tracheobronchial edema

Bronchoscopy was performed and revealed erythema, and sputum retention in the right upper and lower lobe bronchi (Fig. 2). MRSA was again isolated from the bronchial lavage fluid obtained from the right upper and lower lobes whereas the mycobacterial culture was negative. The patient was admitted and treated with intravenous vancomycin (VCM), maintaining the trough concentration between 10 and 15 μg/mL. Blood culture and SARS-CoV-2 PCR performed on admission was both negative. His symptoms and radiological findings improved within a week, and he was discharged after 2 weeks of VCM and switched to oral TMP/SMX for a further 4 weeks. In addition, low-dose erythromycin (200 mg twice daily) was initiated aiming for a local immunoregulatory effect.

**Fig. 3:**
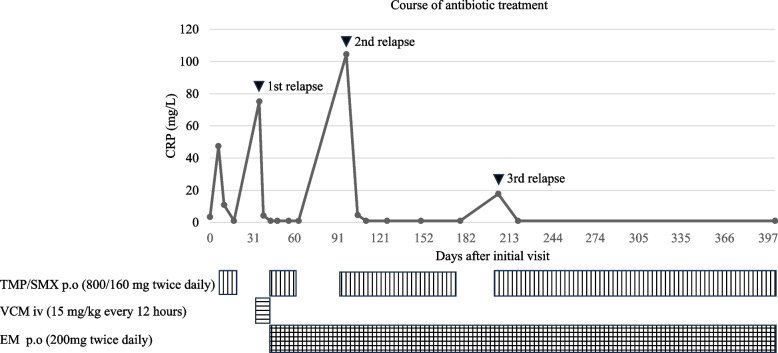
Clinical course observed in this case. Abbrevations. CRP: C-reactive protein, TMP/SMX: trimethoprim/sulfamethoxazole, VCM: vancomycin, EM: erythromycin

However, the patient experienced a second relapse 2 weeks after discontinuing TMP/SMX. Complication of immune deficiency was considered, but HIV antibody was negative, and WBC and immunoglobulin levels were both within the normal range. The MRSA isolate had the same drug susceptibility pattern as that from the first sputum culture, and TMP/SMX was restarted and continued for 3 months, followed by a third relapse after discontinuation of TMP/SMX. MRSA was again detected on culture of bronchial lavage fluid. Confirming that the drug susceptibility pattern of MRSA remained the same, the patient was treated with an extended 6-month course of TMP/SMX (Fig. 3), and achieved infection control. A follow-up CT six months after discontinuation of antibiotics showed improvement in bronchial wall thickening with a slight residual granular shadow, however, the disease has not relapsed for more than one year.

We performed whole-genome sequencing of the first isolated strain, TUM20898. Genomic DNA was extracted with EZ-Beads (Promega K.K., Tokyo, Japan) using the bead-beating method, and then processed with a combination of magLEAD 6gC and magDEA Dx SV (Precision System Science, Chiba, Japan). DNA library preparation and sequencing were performed using Illumina DNA Prep (Illumina, Inc., San Diego, CA, USA) and the Illumina MiSeq platform (Illumina) for paired-end reads of 300 bp using the MiSeq reagent kit v3 600-cycle kit (Illumina). Genome assembly and downstream analysis of sequence data were performed as described previously [2]. The genome analysis revealed that the MRSA strain was member of sequence type (ST)1/staphylococcal cassette chromosome mec (SCC*mec*) type IVa, a clone that is rapidly spreading in Japan. The other genome characteristics and drug susceptibility of the MRSA isolate are shown in Table 1.

## Discussion and conclusions

Although MRSA respiratory tract infections are generally noted for their severity in acute settings, MRSA can also establish long-term colonization in patients with bronchiectasis, including those with cystic fibrosis (CF) [3], affecting long-term prognosis [4]. Long-term management of MRSA infections in outpatient settings can be challenging because of the limited choice of oral antimicrobial agents and their potential adverse effects. Oral linezolid (LZD) is a potential option, however, long-term use of LZD requires caution due to adverse effects such as thrombocytopenia, and ocular and peripheral neuropathy. TMP/SMX is a treatment option for some MRSA infections, such as device-related osteoarticular infections [5]. In addition, TMP/SMX, with or without rifampin, is used in the management of MRSA in patients with CF [6]. In this case, we considered alternative treatments at each relapse, but opted to continue TMP/SMX for 6 months after the third relapse, given its effectiveness and the patient's tolerance, which led to achieving successful control of the condition.

Several factors may have contributed to the development of the disease in this patient. From a host perspective, diabetes and current cigarette smoking status may have contributed to the acquisition of MRSA infection by dysregulating the host immune system. In addition, smoking may have enhanced biofilm formation leading to the persistence of the infection [7]. Furthermore, although no significant structural alterations were observed on CT, underlying mild COPD and a history of pneumonia indicate possible impairment of airway barriers, which may have contributed to MRSA colonization.

With respect to the pathogen, whole-genome sequencing revealed the MLST and SCC*mec* types of the isolates were ST1 with SCC*mec* type IVa, which is one of the dominant clones increasingly detected on blood culture in Japan [1]. This clone has gained attention owing to its recent rapid spread, which has been confirmed by a molecular epidemiological study analyzing single nucleotide polymorphisms [8]. This clone is characterized by the absence of known toxins such as Panton-Valentine leukocidin (PVL) and toxic shock syndrome toxin-1 (TSST-1) [8], which is different from USA400, a ST1 PVL-positive MRSA, which was once prevalent in North America [9, 10]. Another characteristic of this clone is that it possesses collagen adhesin gene (*cna*) [8]. Collagen adhesin (cna) is a cell surface protein that binds to the host extracellular matrix proteins and contribute to biofilm formation. Also, cna can bind to complement protein C1q and inhibit the classical complement activation pathway [11]. In clinical settings, cna is a virulence factor in septic arthritis, where the strength of adhesion to collagen correlates with disease pathogenesis [12, 13]. In addition, *cna*-positive strains have been reported to be associated with bloodstream infections, suggesting that it contributes to bacterial dissemination [14]. This virulence trait also appears to contribute to persistence in the host and may have played a role in the clinical course observed in this case. However, further accumulation of cases and research are warranted to elucidate whether this rapidly spreading clone is related to the refractory course of infection observed in this case.

This rare case of relapsing refractory MRSA bronchopneumonia caused by CA-MRSA ST1/SCC*mec* IVa was controlled by an extended course of oral TMP/SMX. Future studies are required to determine whether the clinical course in this case is typical of that of other cases of infection with this clone, which is rapidly emerging in Japan.

## Data Availability

The draft genome sequence of TUM20898 was deposited in DDBJ/EMBL/GenBank under the accession number JAWLNV000000000.

## Abbreviations

CA-MRSA: Community-acquired methicillin-resistant *Staphylococcus aureus*
CF: Cystic fibrosis
cna: Collagen adhesin
*cna*: Collagen adhesin gene
COPD: Chronic obstructive pulmonary disease
CRP: C-reactive protein
CT: Computed tomography
LZD: Linezolid
MRSA: Methicillin-resistant *Staphylococcus aureus*
PVL: Panton-Valentine leukocidin
SCC*mec*: Staphylococcal cassette chromosome mec
ST: Sequence type
TMP/SMX: Trimethoprim/sulfamethoxazole
TSST-1: Toxic shock syndrome toxin-1
VCM: Vancomycin; WBC: white blood cell
